# Response of a Specialist Bat to the Loss of a Critical Resource

**DOI:** 10.1371/journal.pone.0028821

**Published:** 2011-12-21

**Authors:** Gloriana Chaverri, Thomas H. Kunz

**Affiliations:** Center for Ecology and Conservation Biology, Department of Biology, Boston University, Boston, Massachusetts, United States of America; University of Western Ontario, Canada

## Abstract

Human activities have negatively impacted many species, particularly those with unique traits that restrict their use of resources and conditions to specific habitats. Unfortunately, few studies have been able to isolate the individual and combined effects of different threats on population persistence in a natural setting, since not all organisms can be associated with discrete habitat features occurring over limited spatial scales. We present the results of a field study that examines the short-term effects of roost loss in a specialist bat using a conspicuous, easily modified resource. We mimicked roost loss in the natural habitat and monitored individuals before and after the perturbation to determine patterns of resource use, spatial movements, and group stability. Our study focused on the disc-winged bat *Thyroptera tricolor*, a species highly morphologically specialized for roosting in the developing furled leaves of members of the order Zingiberales. We found that the number of species used for roosting increased, that home range size increased (before: mean 0.14±SD 0.08 ha; after: 0.73±0.68 ha), and that mean association indices decreased (before: 0.95±0.10; after: 0.77±0.18) once the roosting habitat was removed. These results demonstrate that the removal of roosting resources is associated with a decrease in roost-site preferences or selectivity, an increase in mobility of individuals, and a decrease in social cohesion. These responses may reduce fitness by potentially increasing energetic expenditure, predator exposure, and a decrease in cooperative interactions. Despite these potential risks, individuals never used roost-sites other than developing furled leaves, suggesting an extreme specialization that could ultimately jeopardize the long-term persistence of this species' local populations.

## Introduction

Current rates of habitat loss and climate change caused by human activities have negatively impacted a large number of species across all major biomes [1]. Specialist species appear more vulnerable to these activities as they typically exhibit higher extinction rates relative to generalists [2]. The use of a restricted range of resources or habitats puts specialists at greater risk under environmental disturbance because of an increase in competition with generalists, failure to adapt to changing conditions, and an inability or unwillingness to cross gaps of unsuitable habitats to colonize isolated patches [2], [3]. Moreover, compared to behavioral specialists, or those that select specific items among a pool of available resources, species that exhibit functional specialization are more vulnerable to environmental changes because unique physiological or morphological traits restrict them to only a narrow set of resources and conditions [4], [5]. If these critical resources are depleted or lost, populations will decline because individuals are unable to exploit alternative food items or habitats [6], [7].

Mammals have been severely affected by human activities, particularly those responsible for habitat loss and degradation, with 25% of species for which adequate data are available considered threatened with extinction, and accelerated rates of population decline for at least 50% of mammal species [8]. Unfortunately, a substantial reduction in the geographic range of species not only places these species at greater risk of extinction, but also implies a serious loss of ecosystem services and goods [9]. Of particular concern is the loss of services provided by many species of bats, as they are efficient foragers that consume a large number of potentially destructive insect pests, are effective long-distance seed dispersers and pollinators, and provide a number of valued products such as fertilizers [10]. At least 24% of all known species of bats are under threat from human activities such as the introduction of alien species, hunting, loss of foraging habitat, and loss of roosts [11]. Bats that have specialized on specific food items, habitats, or roosts appear to be at greatest risk [7], [11], [12].

Even though habitat loss and fragmentation, overexploitation, and climate change are known to affect long-term population persistence in bats and other organisms, the effects of these threats on populations remain largely unknown, mostly because of the difficulties involved in isolating their individual and combined effects [13]. This uncertainty is considered as a major drawback in projecting changes in biodiversity and extinction risk, and in the design of effective conservation strategies [14]–[16]. Thus, to isolate the response of organisms to specific changes in their environment, it is often necessary to sample populations before and after an experimental or natural perturbation occurs, and to test for changes in biologically significant parameters [17], [18]. In most experimentally or naturally occurring perturbations, the environment is not completely destroyed but rather modified in such a way that only a subset of resources is removed, depleted, or altered (e.g., [19], [20]). The results of such studies are not only useful in isolating the effect of particular threats, but also because they provide clues toward understanding how the performance of individuals (i.e., growth, survival, reproduction) is affected by specific resources [5], and whether these resources can be considered critical [21]. Notwithstanding, conducting such experiments in a natural setting is often difficult because not all organisms can be associated with discrete, easily identifiable landscape features occurring over limited spatial scales that would be amenable to removal experiments. In fact, resource removal experiments are often at odds with conservation priorities, and tend to be very costly.

Here we present the results of a field study that examines the short-term effects of resource loss in a specialist bat species that uses a discrete, easily identifiable habitat feature that occurs over limited spatial scales and is amenable to experimentation, mainly due to the ease with which it can be temporarily removed. Specifically, we experimentally mimicked the loss of roosting habitat in a natural setting and monitored individuals before and after the perturbation to determine patterns of resource use, spatial behavior, and group stability. We investigated changes in these parameters as they can all be used to assess mortality risks, energy expenditure, and cooperation [22]–[26], and ultimately predict resilience to habitat perturbations. Because the study species is highly specialized for exploiting one type of roost [27], and based on results of previous studies addressing the response of populations to habitat loss or resource depletion (e.g., [20], [28], [29]) and on models of resource selection, such as the optimal foraging theory or optimal diet models [30]–[33], we hypothesize that removing roosting habitat will decrease roost selectivity, increase mobility of individuals while attempting to locate suitable habitat, and increase mortality. To our knowledge, this is the first study to closely monitor the behavior of single individuals after experimentally removing a critical resource in a natural setting.

## Methods

### Study species

Our study focused on Spix's disk-winged bat (*Thyroptera tricolor*), a small (3–4 g), insectivorous species found in lowland Neotropical forests from southern Mexico to southeastern Brazil [34]. *T. tricolor* is morphologically highly specialized for roosting in the developing furled leaves of members of the order Zingiberales (primarily in the genera *Heliconia* and *Calathea*), and may be incapable of using other types of roosts that require gripping with the claws [27]. To attach themselves to the inner sides of the leaves while roosting, individuals use suction disks located on their thumbs and feet [35]. The plants used by this bat species for roosting are typically found in secondary forests and clearings, and there is extensive spatial variation in their density [36]. The furled stage of these leaves is suitable for use by *T. tricolor* for very short periods, ranging from 5 to 31 hours [37], [38]. Because *T. tricolor* is highly habitat specific and incapable of using other types of roosts, its distribution is strongly correlated with the density and distribution of furled leaves.

While there are no published studies specifically addressing roost-site preferences in *T. tricolor*, research suggests that this bat uses some plant species more often than others, and that this species may also prefer to use tubular leaves that meet specific requirements. In south-western Costa Rica, *T. tricolor* has predominantly been found in the rolled leaves of *Heliconia imbricata*, and more rarely in *H. rostrata*, *H. latispatha*, and *Calathea* spp. Despite the large abundance of *H. latispatha* in the region, bats are seldom captured in this plant [37]. In north-eastern Costa Rica, *T. tricolor* is known to use 7 species of plants, including several species of *Heliconia*, *Calathea inocephala*, and *Musa* species. The most commonly used plant species in this region is *H. pogonantha* [38]. In addition to these studies that addressed use of specific plants, field observations of occupied and unoccupied tubular leaves have demonstrated that *T. tricolor* prefers more closed and longer leaves for roosting [37], [38], probably because these leaves may provide enhanced protection from weather or predators compared to more opened tubular leaves.

Although there is little available data on the feeding ecology of *T. tricolor*, studies on their diet [39], echolocation [40], and morphology [41] suggest that this species is primarily a gleaner, feeding mostly on jumping spiders and leafhoppers. Their short, broad wings are well suited for the slow, maneuverable flight necessary for gleaning, but may also set an upper limit as to how far they can fly during nighttime foraging bouts, potentially making them poor dispersers [42]. In fact, our recent findings show that *T. tricolor* exhibits low emigration rates from, and long residence times within, natal territories, coupled with high levels of offspring retention from both sexes within natal groups [43]. Offspring natal philopatry results in the formation of mixed-sex groups composed of up to 14 individuals, which maintain a local distribution and small, overlapping, home ranges [44]. Despite their spatial overlap, groups are highly cohesive for up to 22 months, without immigration of individuals from other groups or emigration of group members [45]. Recent findings show that *T. tricolor* uses social calls to actively recruit group members to roosts [46], suggesting that acoustic communication may play an important role in group cohesion and in the location of roost sites.

### Study sites and sampling of populations

This study was conducted at six sites, Bolivar (8°38′N, 83°04′W), Eduardo (8°41′N, 83°08′W), Desanti (8°36′N, 83°03′W), Suita, Catarata, and Rio (8°41′N, 83°07′W), located in the lowlands of private properties located in southwestern Costa Rica. These research areas consist mainly of dense tropical broad-leaved evergreen lowland forests, with surrounding habitats that include pasturelands, hardwood plantations, agricultural crops, and human settlements. Sites were located near river beds in valley floors, mostly within or immediately adjacent to late-secondary or primary forests. Only one site (Bolivar) was located within a *Gmelina arborea* hardwood plantation. The size and shape of experimental plots within sites were selected based on 1. naturally-occurring dense patches of *Heliconia imbricata* plants surrounded by relatively unsuitable roosting habitats, 2. the presence of a *T. tricolor* group, and 3. the size and shape of the group's home range before the habitat loss experiment. Patches of *H. imbricata* were preferred over those of other plant species because this is the preferred roosting resource of *T. tricolor* in southwestern Costa Rica (G. Chaverri, unpublished data; [37]).

Study plots were sampled for bats before the start of habitat removal experiments to gather baseline data of roost species preferences, patch use, group home range size, and group cohesion. All tubular leaves within the plot were searched, and bats were captured in all identified roosts by pinching the top of the leaf and directing individuals into a cloth-holding bag. All bats were fitted with individually numbered metal wing bands, sexed, aged based on the degree of ossification of the metacarpal-phalange joint [47] and their reproductive condition assessed [48]. All protocols for capturing and handling bats were approved by the Costa Rican government (permit number R-008-2009-OT-CONAGEBIO) and by Boston University's Institutional Animal Care and Use Committee (approval number 02-005).

All sites had one social group that had been captured and/or radiotracked several times before the habitat removal experiment. The size of these groups ranged from 6 to 9 individuals (Table 1). At sites B (Bolivar), S (Suita), and E (Eduardo), we sampled the focal group 6 to 8 times during a period of 4–5 months before experimentally removing the habitat (Table 1). Thus, data collected from this period were treated as the “before” data. At the other three sites, D (Desanti), C (Catarata), and R (Rio), we captured the focal group 0 to 4 times during a period of 2 to 4 months before fitting them with transmitters. These transmitters were attached for 5 to 7 days before removing the habitat (Table 1). Thus, at D, C, and R, the “before” data consisted of observations collected over a few months together with daily data collected after attaching radiotransmitters and before removing roosting resources.

**Table 1:** Sampling protocol for each one of the six study sites.

	Group size		Before		After	Radiotagged	
Site	M	F	RC	RT	RT	M	F
B	7	3	6	0	8	3	2
S	5	2	8	0	7	2	2
E	2	4	6	0	7	2	3
D	3	3	4	5	12	1	3
C	4	2	0	7	9	2	1
R	5	4	3	6	10	3	4

Group size refers to the number of males (M) and females (F) present in each one of the focal groups. Sample size before and after habitat removal refers to the number of days in which bats were located based on roost captures (RC) or radiotelemetry (RT), with the number of males and females that were radiotagged per focal group.

### Habitat loss experiment

To mimic habitat loss, we cut all plants that could potentially be used as roosts in the experimental plots. This includes all species of *Heliconia*, such as *H. imbricata*, *H. latispatha*, *H. irrasa*, and *H. stilesii*; all *Calathea*, including *C. lutea*, and *C. inocephala*; and all *Musa*. Plants were identified based on floral characteristics using field guides [49], [50]. We attempted to remove all plants that were located not only within the home range of the focal group, as determined by the location of roosts during the “before” observations, but also some immediately adjacent plants. Plants were cut at a height of approximately 50 cm; this would guarantee the removal of all leaves that could potentially be used as roosts for the following 2–3 weeks, while securing the long-term survival of plants.

At the time of habitat removal, we first captured the focal group and fitted several bats at sites B, S, and E, with small radiotransmitters (0.25 g, Blackburn Electronics, Nacogdoches, Texas; Table 1). Bats were released at their capture site after removing habitat, and subsequently located at their roosts for as long as the radiotransmitter remained active and attached (Table 1). Bats in sites B, S, and E were located in their roosts after removing habitat a total of 7 to 8 days. Data collected during this period were treated as the “after” data. At the remaining sites, D, C, and R, we fitted 3 to 7 individuals with radiotransmitters that lasted 16–17 days (0.20 g, model A2412, Advanced Telemetry Systems, Isanti, Minnesota; Table 1). At these sites, bats were captured, fitted with transmitters, and then released at the same location and tracked for a few days before experimentally removing potential roosts. After habitat removal, bats were located in their roosts for 9 to 12 days (Table 1). During this period, data were treated as the “after” removal of roosting habitats.

### Data analysis

Before and after habitat removal, we collected data on plant species used for roosting, location of roosts to estimate home range size, patch use, and group composition to measure group stability. To explore differences in the number of species used before and after habitat removal, we used the rarefaction method. This method is used for estimating the number of species expected in a random sample of individuals, thus allowing us to standardize our results to a common sample size [51]. Thus, in our study, rarefaction curves allow us to determine the estimated number of plant species used by the bats if sampling during the “before” and “after” trials would have been the same. Rarefaction curves were generated separately for both trials using EcoSim 7 [52] and plotted together for further comparison.

All roosts recorded during the before and after trials were located using a Global Positioning System (eTrex, Garmin International Inc., Olathe, Kansas). We then calculated the size of the roosting home range for each individual before and after habitat removal by drawing 100 percent Minimum Convex Polygons. For this purpose we used the Home Range Extension [53] in ArcGIS 9.2 (Environmental Systems Research Institute, Redlands, California). We log transformed measures of home range size to meet assumptions of normality. Data were then analyzed using a linear mixed-effects model using the restricted maximum likelihood method in SPSS version 17.0 (SPSS Inc., Chicago, Illinois) to determine if there were differences in home range size among the before and after trials while accounting for the effect of site and the possibility that home range size may be correlated among group members. Thus, in the model, trial was treated as a fixed effect, while site was treated as a random effect. We also ran a general linear model in SPSS, with trial and site as fixed factors, to determine if there were significant differences in home range size between trials within sites, using Bonferroni correction for multiple pairwise comparisons.

After establishing the location of bats before and after the removal of all potential roosts, we also mapped the study site using ArcGIS, noting the specific locations of habitat patches, roosts used by the focal group during the before and after trials, and location of other social groups. The size and shape of each site were selected according to the movement of bats before and after the removal of roosts, such that our maps and analyses of patch use encompass all potentially available resources in the area based on realistic results of animal movements. Based on these maps, we determined how bats were using roosting resources before and after habitat removal by comparing the observed data with five predictive models: 1) patchy resource, 2) preferred species, 3) largest patch, 4) nearest patch, and 5) unoccupied site. To avoid problems with pseudoreplication, we tested these models using data from groups and not individuals. For the *patchy resource* model, we predicted that bats would use clumped roosts, defined as *Heliconia* spp. or *Calathea* spp. plants located close to each other (i.e., 1–3 m) in an area greater than 100 m^2^. For the *preferred species* model, we predicted that bats would use *H. imbricata*, their preferred plants species in the study area, as roost sites. We also predicted that bats would use the largest patch in the area (*largest patch* model), and that they would use the patch closest to the group's core home range (*nearest patch* model). Finally, we predicted that roosts used by the focal group would be located in a patch that was not occupied by another group. All data were classified as yes or no, depending on whether groups were using a roost according to the proposed models. Observed results of these five models were tested against the expectation that bats would primarily (i.e., 99% of the time) use *H. imbricata* roosts located in the largest and closest unoccupied patches. Less stringent expectations (e.g., 95% of the time) resulted in similar trends. We compared our field observations with these expected values based on chi-squared goodness of fit tests [54].

To determine if group stability varied as a result of habitat removal, we first calculated the simple ratio association index [55], [56] for each individual and trial (before and after). This index estimates the proportion of time that two individuals (or dyad) spent in association, and ranges from 0 (no association) to one. In this analysis, a dyad was considered to be associating if individuals were captured or tracked at the same roost at the same time. The simple ratio association index was calculated as X/(X+Y_AB_+Y_A_+Y_B_), where X is the number of observations during which bat A and bat B were observed together in the same roost, Y_AB_ is the number of observation periods during which A and B were observed in separate groups, Y_A_ is the number of observation periods during which only A was observed, and Y_B_ the number of observations in which only B was observed. Analyses of associations were performed in SOCPROG version 2.4 [57]. To test for the effect of habitat removal on group stability, we used Mantel tests with 10,000 permutations in SOCPROG to compare differences in association indices before and after removing habitat for the six study sites independently. In addition, to test the correlation between home range size and association indices, we ran a linear regression with standard errors clustered across sites and individuals [58] in Stata/SE 10.0 (StataCorp, College Station, Texas).

## Results

### Plants used for roosting

**Figure 1:**
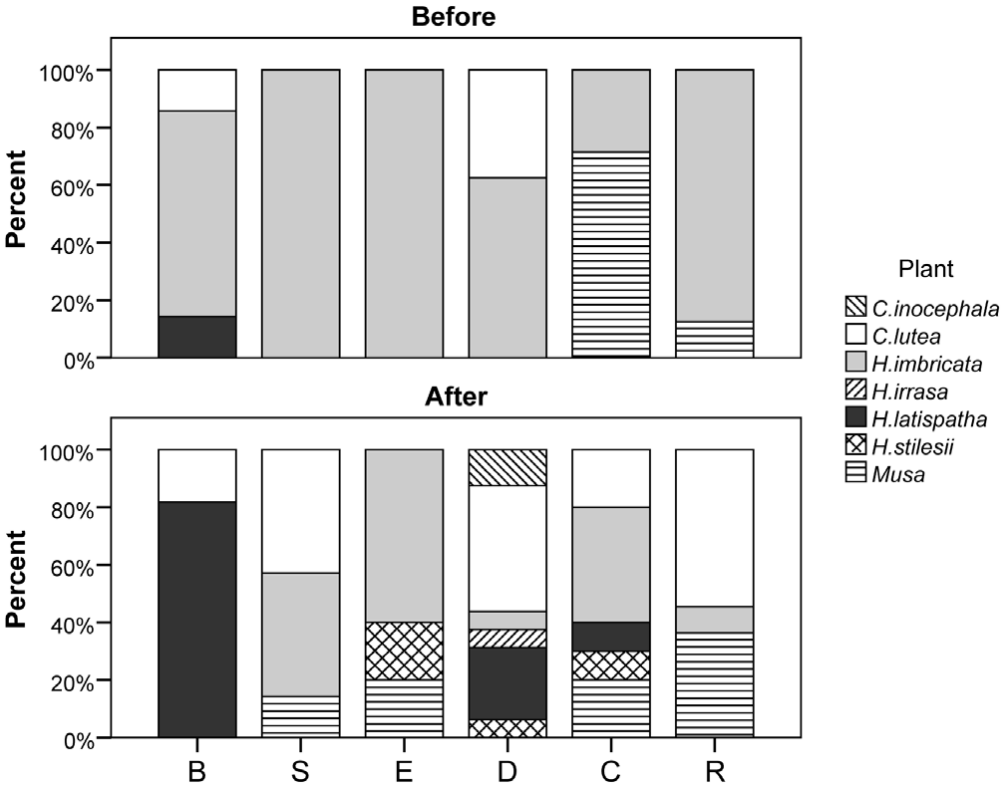
Plant species used for roosting before and after removing habitat. Number of observations per site per trial is indicated within the corresponding bars.

**Figure 2:**
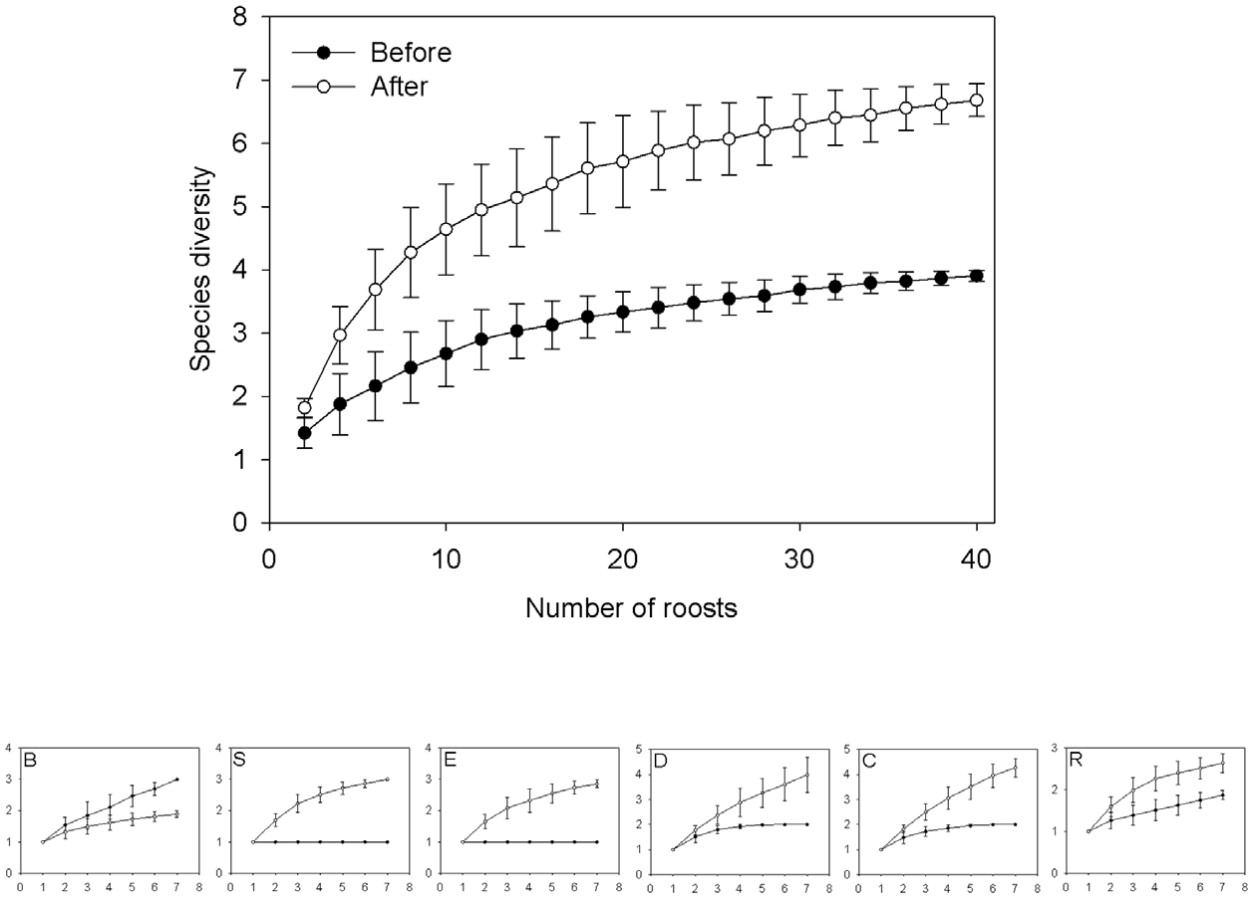
Rarefaction curves for plant species used as roosts before and after habitat removal for all sites combined (upper graph) and for each one of the six sites (lower graphs).

Before their roost habitat was removed, at most sites *T. tricolor* primarily used *H. imbricata* for roosting (Fig. 1). Some groups also used other species such as *C. lutea*, *H. latispatha*, and *Musa* sp. The only site in which bats predominantly used a species other than *H. imbricata* before removing habitat was C, where the majority of roosts were recorded in *Musa* sp. For all sites, individuals continued to use *H. imbricata* in the after trial, only not as frequently as they did during the before trial. There was no apparent preferred plant species for roosting during the after trial. Individuals at different sites used a minimum of one and a maximum of three plant species for roosting during the before trial (*n* = 44), while in the after trial individuals used a minimum of two and a maximum of six species per site (*n* = 65). Rarefaction curves for all sites combined show that the richness of plant species used for roosting increased once the habitat was removed (Fig. 2). With the exception of site B, individuals at all sites used a greater diversity of plants for roosting once their habitat had been removed.

During the before trials, bats typically used furled leaves within their preferred size range, height, and inclination. In the after trial, however, we observed bats using leaves that were much wider than usual. We also recorded individuals in smaller plants (e.g., openings of tubular leaves were approximately 1 magl), and we also found individuals roosting one day in a tubular leaf whose inclination approached a 45° angle. In our 436 bat-days of observation, we never recorded individuals roosting in any structure other than tubular leaves.

### Home range size and patch use

**Figure 3:**
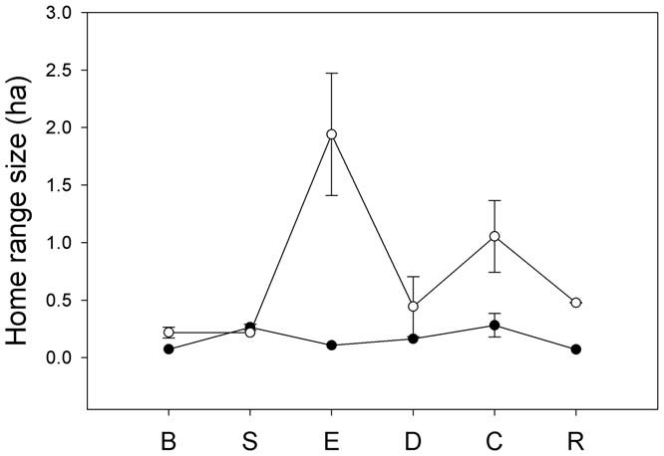
Mean home range size (in hectares) before (filled circles) and after (open circles) removing habitat at the six study sites. Error bars represent mean±SD.

Overall, *T. tricolor* showed a significant increase in home range size after their habitat was removed (before: mean 0.14±SD 0.08 ha; after: 0.73±0.68 ha; F_1,46_ = 85.67, P<0.001). Only 5 out of 27 individuals exhibited a decrease in home range size after habitat removal. A significant increase in home range size during the after trial was observed in sites E, C, and R (Fig. 3). In all other sites, except S, there was also a mean increase in home range size between the before and after trials. The smallest difference in home range size between trials was observed in S, where it decreased by 0.04 ha after removing habitat. The largest difference in home range size between trials was observed in E, where average size increased from 0.10 to 1.94 ha (Fig. 3).

**Figure 4:**
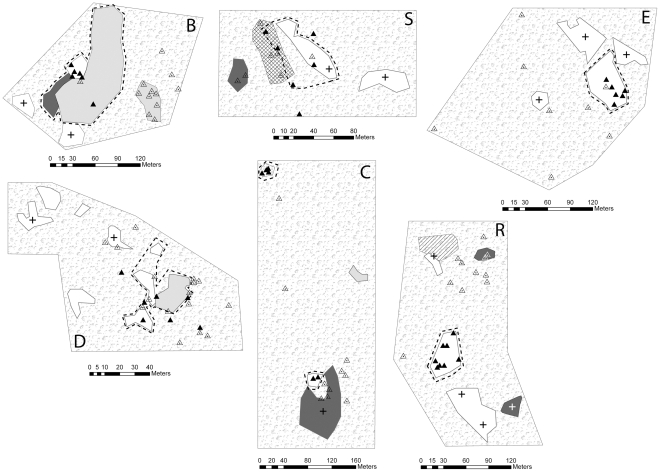
Maps of each one of the six study sites showing patches of plant species used by *T. tricolor* for roosting (white: *H. imbricata*, light grey: *H. latispatha*, dark grey: *C. lutea*, stippled: *Musa* sp., simple-hatched: mixed *C. lutea* and *H. latispatha*, cross-hatched: mixed *C. lutea* and *H. imbricata*). Dashed line indicates the removed area. Filled and opened triangles represent location of roosts of target group before and after habitat removal, respectively. Crosses represent location of other groups.

Before habitat was removed, all focal groups were consistently captured in *H. imbricata* patches that ranged in size from approximately 100 to 2,500 m^2^ (Fig. 4). These patches were often surrounded by a few sparse plants of *H. imbricata* and by dense patches of *C. lutea* and *H. latispatha*, which were occasionally used by the focal group as roosts. At all sites, surrounding dense habitat patches of *H. imbricata* were typically occupied by other groups, and were never used either before or after habitat removal by the focal group. Bats returned to their original habitat patch as soon as regenerating plants produced suitable furled leaves, approximately 3–4 weeks after plants were cut.

**Figure 5:**
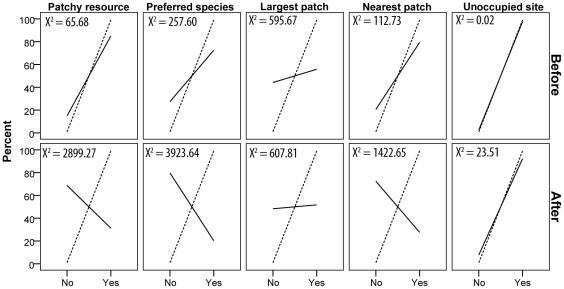
Comparison of observed (solid line) and expected (dashed line) frequency of resource use before and after habitat removal, with results from the chi-square goodness of fit test. Models tested were those that address whether bats used 1) a patchy roosting resource, 2) their preferred plant species (*H. imbricata*), 3) the largest patch in the area, 4) the patch closest to the group's core home range, and 5) a roost located in a site that was not occupied by another group.

Results of our models of resource use show that observed and expected values of the *unoccupied site* model provided the best fit to the data either before or after habitat removal, as demonstrated by the low chi-square values (Fig. 5). Models also indicate that before habitat removal, bats predominantly used patchy roosting resources of their preferred plant species near their core home range. This trend changed considerably after the habitat was removed, as bats predominantly used more scattered and farther roosting resources of other non-preferred species.

### Group stability and mortality

**Figure 6:**
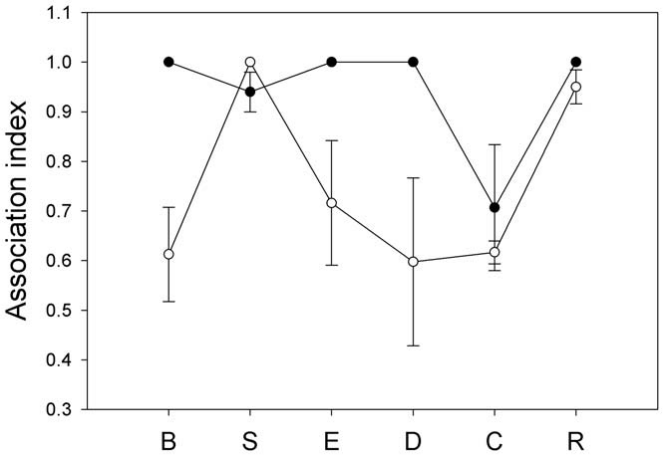
Mean association indices before (filled circles) and after (open circles) removing habitat at the six study sites. Error bars represent mean±SD.

Group composition was stable both before and after habitat removal, with dyadic association indices ranging from 0.35 to 1. The site with the lowest average association index during the before trial was C (0.70±0.12; Fig. 6). In the before trials, groups sampled at sites B, E, D, and R showed no change in the composition of groups. Thus, all individuals at these sites were observed together during the period before removing habitat. Overall, a significant decrease in mean association was observed after removing roosting habitat (before: 0.95±0.10; after: 0.77±0.18). Significant differences in mean association between trials were observed in B, E, and R (Mantel test with 10,000 permutations: p<0.05). The smallest decrease in mean association was observed in R, while the largest decrease after removing roosting habitat was observed in D. Regression analysis shows that association indices were significantly correlated with home range size (R^2^ = 0.13, F_1,26_ = 15.77, P<0.001).

While changes in group composition were mostly attributed to individuals from the same group roosting in separate leaves at the same time, mortality also had an important effect on measures of group stability. In site B, a radiotagged male was found dead on top of a leaf one day after attaching the radiotransmitter, which suggests that this individual was unable to tolerate the additional mass. In fact, radiotags often exceeded by 1 or 2% the recommended 5% relationship between the mass of radiotags and the mass of bats [59]. Another male died in site E 6 days after the transmitter had been attached and the habitat was removed, and a female in D died 7 days after removing the habitat and 11 after attaching the radiotransmitter. These latter individuals were found dead inside furled leaves, and the time elapsed since the attachment of transmitters suggests that the extra mass may not have been primarily responsible for their deaths. In addition, one day after removing habitat in site C we were unable to track a tagged female despite efforts to extend our sampling area, suggesting either a malfunction of the transmitter, permanent departure of the bat to a relatively distant area, or predation. Finally, a lactating non-tagged juvenile that had been roosting with his tagged mother since his birth in site B, disappeared by the end of the experiment and before radiotransmitters were collected.

## Discussion

### Behavioral response to the loss of roosting resources

The results of our study demonstrate that, as predicted, the removal of roosting resources within habitat patches had a considerable effect on the behavior of individuals. In particular, we found that habitat removal was associated to a decrease in roost-site preferences and/or species selectivity, and a change in resource use from nearby, patchy resources to selection of more scattered and distant roost sites. This resulted in an increase in the mobility of individuals, and a decrease in social cohesion. These behavioral responses were expected based on well-developed models of resource selection, such as the optimal foraging theory or optimal diet models, and on findings from a diversity of studies addressing the correlation between spatial and social dynamics and resource abundance and distribution.

In terms of resource preferences, theoretical models suggest that animals should use a greater diversity of food items, even those of low quality, when food is scarce, whereas individuals should specialize on high-quality items as food becomes more abundant [30]–[33]. Many empirical studies on a wide diversity of taxa support the predictions of these foraging models (e.g., [60]–[62]), and studies also show that these models can also be applied to other resources [20]. Our study is the first to expand the predictions of these models to roost-site selection in bats, as results demonstrate that loss of preferred roosting resources increases the diversity of sites used by individuals.

Because home range size is constrained by the need of individuals to obtain sufficient resources in the smallest area possible, the quality of the resources available to an animal within the portion of habitat actually used is a major correlate of its ranging behavior [63]. In this regard, several studies confirm an increase in the size of an individual's home range in response to insufficient resources (e.g., [64]–[67]). Our results show that bats increased the size of the areas used for roosting when resources were depleted from a group's home range. Interestingly, while many other patches of *H. imbicata* were typically located near the focal group's home range, bats rarely occupied these sites after their patch was removed. These patches were always occupied by another group, which suggests that resident groups may defend patches which are then unavailable to other individuals.

The stability of social groups is affected by a number of intrinsic and extrinsic factors, including aggression and cooperation levels, relatedness among group members, parasite load, and group size [68]–[73]. Home range size can also influence group stability if the use of large areas implies higher rates of mortality due to an increase in energetic demands and from an increased exposure to predators [22], [23], or if long-distance movements hinder the transmission of signals used to coordinate social cohesion [74]. Thus, a decrease in group stability after habitat removal was expected given the general increase in home range size and its potential effect on mortality rates, and the fact that *T. tricolor* relies on acoustic communication to convey information about roost location [46], [75].

### Loss of roosts and fitness consequences

Diurnal roosts are a valuable resource for bats because they protect individuals from temperature extremes and predators [24], [76], and are one of the most important venues for social interactions [77]–[79]. It is no surprise then that individuals exhibit an immediate and considerable behavioral response to the loss of roosting resources in order to find alternative roosting sites. Notwithstanding, we hypothesize that this response may also be responsible for a decrease in the survival of individuals. In this respect, our results suggest that individuals must fly over larger areas possibly for longer periods of time to locate suitable roost sites after the loss of roosting resources in their habitat patch, which most likely increases daily energetic expenditure [80]. Significant increases in energetic expenditure could result in greater mortality rates if individuals are unable to compensate this energy loss by increasing food intake [81]. In addition, greater energetic expenditure during lactation could result in severe limitations to energy allocation towards dependent young and a subsequent increase in levels of juvenile mortality [82]. An increase in search time after the loss of roost-sites also means that individuals could suffer from greater rates of predation [83], and that the use of suboptimal roosts (i.e., more opened leaves) could also render individuals more vulnerable to predators that rely on visual cues to locate prey and to extreme fluctuations in environmental conditions.

In addition to greater mortality rates due to increased energetic expenditure and predator vulnerability, the loss of roosting resources could also have detrimental effects on fitness due to changes in cooperative interactions. Many species of bats rely on some form of cooperative behavior to increase young survival, defend feeding resources, share food, or locate roost sites [46], [84]. Because reduced encounter rates are known to hinder reciprocation [26], we speculate that a decrease in group cohesion due to changes in the availability of roosting resources and/or changes in home range size could hinder cooperation among group members. If individuals rely on information transfer to locate clumped and unpredictable resources, a decrease in cooperation rates among group members could reduce resource acquisition efficiency, significantly increasing search times and the risks and costs incurred during this process.

### Behavioral and functional specializations and population persistence

Our findings of roost-site selection before and after habitat removal suggest that *T. tricolor* exhibit behavioral specializations in which individuals appear to select specific plant species for roosting but may use others when the former are unavailable. Many studies have shown that bats generally select roosts that provide a set of conditions that favor energetic savings and predator avoidance, even though they may be well suited to roost at a greater range of sites and are known to do so if ideal roosts are not available [24], [85], [86]. In addition to our observations of behavioral specialization, and based on the lack of use of other structures beside furled leaves despite their sudden disappearance from habitat patches and their relative scarcity at some sites, our study confirms that morphological specializations in *T. tricolor* may restrict it predominantly (or exclusively) to the use of furled leaves. At least 5 other species of bats have adhesive organs on their wrists and ankles [87], [88], and some appear to roost also primarily in the smooth surfaces of developing furled leaves [89]. This suggests that they could be equally restricted in their use of roost sites, and may exhibit similar behavioral responses to the loss of roosting resources as those observed in *T. tricolor*.

Most research to date shows that functional specializations may place species at greater risk of extinction than behavioral specializations [4], [5]. While the adhesive organs of sucker-footed and disc-winged bats have allowed them to exploit a ubiquitous resource in many tropical habitats and avoid competition with other species for roost sites, their extreme reliance on this type of roost could also render them extremely vulnerable to major changes in plant availability, particularly if there are high costs associated with long-distance movements that would limit colonization of alternative patches. Foliage gleaners such as *T. tricolor* [39] forage in cluttered spaces and have wing morphologies that increase manoeuvrability at the expense of aerodynamic efficiency and speed. Thus, compared to insectivorous species that forage above the canopy, foliage gleaners probably incur greater energetic expenditure during long-distance flight than bats specialized for aerial hunting [41]. Long-distance movements could be further constrained in species such as *T. tricolor* that rely on a patchily distributed resource because the landscape provides little connectivity among habitat patches. This problem could be aggravated if the species in question exhibits territorial behavior, as this further reduces the number of resources available to dispersing individuals.

### Conclusions

Understanding the effects of disturbances on individuals, populations, and species, makes it possible to successfully guide conservation efforts and manage ecological resources. Unfortunately, given the technical difficulties, financial cost, and conflicts with conservation-related initiatives inherent to experimental perturbations of natural habitats, isolating the response of organisms to specific changes in their environment has proven challenging. Behavioral strategies are leading indicators for conservation purposes as they are linked to fitness and hence can be used to forecast population dynamics, and they exhibit immediate changes to altered environmental conditions [90]. Our study shows that experimental perturbations to gauge the behavioral response of a specialist mammal to specific changes in its environment and to predict the effect of resource loss on demographic parameters, such as dispersal and mortality, are feasible, particularly if the study species relies on conspicuous and easily modifiable resources. Our study also demonstrates that the loss of a critical resource in a specialist bat elicited behavioral responses that may reduce fitness by potentially increasing energetic expenditure, predator exposure, and a decrease in cooperative interactions. Despite these potential risks, and the fact that bats faced an immediate and considerable loss of a critical resource, individuals never used alternative roost-sites, suggesting an extreme specialization that could ultimately jeopardize the long-term persistence of this species' local populations.
